# ATP-binding cassette transporter expression is widely dysregulated in frontotemporal dementia with TDP-43 inclusions

**DOI:** 10.3389/fnmol.2022.1043127

**Published:** 2022-11-01

**Authors:** Jared S. Katzeff, Hiu Chuen Lok, Surabhi Bhatia, YuHong Fu, Glenda M. Halliday, Woojin Scott Kim

**Affiliations:** Brain and Mind Centre & School of Medical Sciences, The University of Sydney, Sydney, NSW, Australia

**Keywords:** frontotemporal dementia, ATP-binding cassette subfamily A, ABCA transporters, lipids, TDP-43, neurodegeneration

## Abstract

The human brain is highly enriched in lipids and increasing evidence indicates that dysregulation of lipids in the brain is associated with neurodegeneration. ATP-binding cassette subfamily A (ABCA) transporters control the movement of lipids across cellular membranes and are implicated in a number of neurodegenerative diseases. However, very little is known about the role of ABCA transporters in frontotemporal lobar degeneration with TDP-43 inclusions (FTLD-TDP), which is a common form of younger-onset dementia. We therefore undertook a comprehensive analysis of the expression of ABCA transporters (ABCA1–13) in five key brain regions (amygdala, inferior temporal cortex, superior frontal cortex, cerebellum and parietal cortex) in FTLD-TDP and controls. We found that the expression of ABCA2, ABCA3, ABCA4, ABCA7, ABCA9, ABCA10 and ABCA13 was significantly altered in FTLD-TDP in a region-specific manner. In addition, the expression of ABCA transporters correlated specifically to different neural markers and TARDBP. These results suggest substantial dysregulation of ABCA transporters and lipid metabolism in FTLD-TDP and these changes are associated with neuroinflammation.

## Introduction

ATP-binding cassette subfamily A (ABCA) consist of 12 members named ABCA1-13, with ABCA11 being a pseudogene. The primary role of ABCA transporters is transport of lipids, such as cholesterol and phospholipids, across cellular membranes (Piehler et al., 2012). ABCA transporters have been implicated in pathologies including ABCA1 in Tangier’s disease (Brooks-Wilson et al., 1999), ABCA4 in Stargardt macular dystrophy (Allikmets et al., 1997) and ABCA12 in harlequin-type ichthyosis (Kelsell et al., 2005). The role of lipid transporters in the brain is important due to the high lipid content of the brain, with approximately 40% of grey matter and 65% of white matter consisting of lipids (Obrien and Sampson, 1965). Dysregulation of ABCA transporters expression is associated with various neurodegenerative diseases (Katzeff and Kim, 2021). ABCA1, ABCA2 and ABCA7 have been linked to Alzheimer’s disease (AD). *ABCA7* gene variants are the fourth highest genetic risk factor linked to late-onset AD (Hollingworth et al., 2011), while genetic variants of *ABCA1* and *ABCA2* are known risk modulators of AD (Mace et al., 2005; Lupton et al., 2014). As well as lipid dysregulation, the pathological role of ABCA7 is related to amyloid-β clearance and phagocytosis (Fu et al., 2016; Sakae et al., 2016).

Inclusions of TAR DNA-binding protein-43 (TDP-43) have been estimated to occur in 19–57% of AD cases (Amador-Ortiz et al., 2007; Josephs et al., 2014). However, the expression of ABCA transporters in other TDP-43 pathologies such as frontotemporal lobar degeneration with TDP-43 inclusions (FTLD-TDP), which accounts for 32–54% of total FTLD cases, is currently unknown. FTLD-TDP is characterized by aberrant nucleocytoplasmic transport of TDP-43, which results in deposition of phosphorylated TDP-43 in the cytoplasm. Of interest, TDP-43, which is encoded by the *TARDBP* gene, has extensive functions including transcription, translation and mRNA trafficking, and alterations in its physiological functions are known to associate with mitochondrial dysfunction and neuroinflammation, the latter being increasingly recognized as a hallmark of FTLD-TDP pathology (Bevan-Jones et al., 2020).

The expression of ABCA transporters is known to modulate neuroinflammation, which has been shown to have a close association with protein aggregation in FTLD-TDP, as indicated by the presence of activated microglia in regions with TDP-43 pathology (Trageser et al., 2019). Indeed, it has been reported that microglia from *ABCA1* knockout mice exhibit a heighted pro-inflammatory response (Karasinska et al., 2013). In addition, *ABCA7* knockout or haploinsufficiency in AD hinders amyloid-β clearance, suggesting that ABCA7 expression has a neuroprotective role and modulates microglial responses in neurodegeneration (Fu et al., 2016; Aikawa et al., 2018).

In the current study, the transcriptional levels of all ABCA transporters, common neural cell markers and TARDBP were measured in brain regions progressively affected in FTLD-TDP. TARDBP was measured to determine if there was a correlation between TDP-43 pathology and dysregulation of ABCA transporter expression. Firstly, ABCA expression changes in FTLD-TDP compared to controls were determined, followed by correlating these expression changes with neural subtype makers and TARDBP expression.

## Materials and methods

### Human brain tissues

Fresh-frozen post-mortem brain tissue samples were obtained with consent from the Sydney Brain Bank and NSW Brain Tissue Resource Centre. All brain donors underwent standardized assessments in life and standardized neuropathological examination, and met current consensus diagnostic criteria for FTLD-TDP (Cairns et al., 2007; Mackenzie et al., 2010) or no significant neuropathology (controls; Hyman et al., 2012; Montine et al., 2012). Tissue samples from the amygdala (earliest affected), inferior temporal and superior frontal cortices (next affected), cerebellum and finally parietal cortex (Rabinovici and Miller, 2010) of 10 FTLD-TDP sporadic cases (5 male, 5 female) and 11 controls (5 male, 6 female without neurological, psychiatric or neuropathological disease) were used in this study (Table 1). The 10 FTLD-TDP cases were previously confirmed as having TDP-43 pathology (Tan et al., 2013). The mean age of the two groups were 72.9 ± 13.0 and 79.5 ± 12.1 years, respectively. Ethics approval for the study was from the University of New South Wales Human Research Ethics (approval number: HC15789).

**Table 1:** Demographic information of cases used in this study.

ID	Case	Age	Sex	PMI (h)	Disease dura. (y)	Brain pathol.
1	FTLD	66	M	39	2	TDP-43
2	FTLD	62	M	15	3	TDP-43
3	FTLD	72	F	25	1	TDP-43
4	FTLD	61	M	37	2.5	TDP-43
5	FTLD	65	F	22	5	TDP-43
6	FTLD	84	F	17	8	TDP-43
7	FTLD	60	M	28	3	TDP-43
8	FTLD	99	F	13	14	TDP-43
9	FTLD	86	F	25	8	TDP-43
10	FTLD	74	M	20	7	TDP-43
11	Control	85	F	23	N/A	N/A
12	Control	79	M	8	N/A	N/A
13	Control	89	F	23	N/A	N/A
14	Control	101	F	9	N/A	N/A
15	Control	84	M	9	N/A	N/A
16	Control	93	F	15	N/A	N/A
17	Control	74	M	10	N/A	N/A
18	Control	63	M	24	N/A	N/A
19	Control	66	M	23	N/A	N/A
20	Control	74	F	20	N/A	N/A
21	Control	67	F	15	N/A	N/A

### Protein extraction

Tris-buffered saline (TBS) and SDS-soluble proteins were serially extracted from 100 mg of fresh-frozen brain tissues, as previously described (Murphy et al., 2013). Briefly, tissues were homogenized in 10 volumes of TBS homogenization buffer (20 mM Tris, 150 mM NaCl, pH 7.4, 5 mM EDTA, 0.02% sodium azide) containing protease inhibitor cocktail (Roche) using Qiagen TissueLyser (3 × 30 s, 30 Hz cycles), followed by centrifugation at 100,000 *g* for 1 h at 4°C, with supernatant collected as TBS-soluble fraction. The pellet was resuspended in SDS solubilization buffer (TBS homogenization buffer containing 5% SDS) using 3 × 30 sec, 30 Hz cycles with TissueLyser, and centrifuged at 100,000 *g* for 30 min at 25°C, with supernatant collected as SDS-soluble fraction. Protein concentration was measured using a bicinchoninic acid assay (Pierce BCA Protein Assay Kit) following the manufacturer’s instructions.

### Western blotting

Western blotting was carried out as previously described (Phan et al., 2020). Protein lysates (10 μg) were heated with sample buffer (3.2% SDS, 32% glycerol, 0.16% bromophenol blue, 100 mM Tris–HCl, pH 6.8, 8% 2-mercaptoethanol). They were then electrophoresed on Criterion Stain-free 4–20% SDS-PAGE gels (Bio-Rad) and transferred onto nitrocellulose membranes at 100 volts for 30 min. The membranes were blocked with TBS containing 5% nonfat dry milk and probed overnight at 4°C with TDP-43 antibody (Proteintech, 10,782-2-AP, 1:2,000) and β-actin (Abcam, ab6276, 1:10,000). They were then washed three times in TBS containing 0.1% Tween 20 and incubated with horseradish peroxidase-conjugated secondary antibodies for 2 h at room temperature. Signals were detected using enhanced chemiluminescence and Gel Doc System (Bio-Rad).

### Immunohistochemistry

Immunohistochemistry of FTLD-TDP superior frontal cortex was carried out as previously described (Phan et al., 2021). Briefly, formalin-fixed, paraffin-embedded sections (10 μm) were deparaffinized in xylene and rehydrated through graded ethanol, followed by antigen retrieval with citrate buffer (pH 6.0) using a pressure cooker (Aptum Bio Retriever 2,100, Aptum Biologics Ltd., United Kingdom) at a peak temperature of ~121°C and gradually cooling to room temperature. Endogenous peroxidase was blocked with 1% hydrogen peroxide in 50% ethanol. Sections were probed with TDP-43 antibody (Proteintech, 10,782-2-AP, 1:400) and NeuN antibody (Biolegend, SIG-39860, 1:100), washed with PBS and incubated with the corresponding secondary antibodies (Thermo Fisher Scientific, A-10042 and A-31571, 1:250) and 4′,6-diamidino-2-phenylindole DAPI (Sigma-Aldrich, D9542, 1 mg/ml). The slides were treated with 70% Sudan Black for 30 min and 10 mM CuSO4 in 50 mM ammonium acetate buffer (pH 5.0) to quench auto-fluorescence signals prior to cover-slipping with anti-fade fluorescence mounting medium (DAKO, S3023) and then sealed with nail polish. Negative controls (without primary antibodies or secondary antibodies) were performed for each immunohistochemistry run, and no signals were detected in each case.

### Microscopy imaging

For immunohistochemistry, stained sections were scanned using an Olympus VS120 Slide Scanner with the same focus and exposure settings. For immunofluorescence, multiple sections were examined and representative images were captured with a Nikon C2 confocal microscope and associated Nikon NIS Elements software (version 4.60). Images were adjusted for contrast and converted to TIFF format on Fiji software (ImageJ version 2.0.0-rc-69/1.52p).

### RNA isolation, reverse transcription and quantitative PCR

RNA was isolated using TRI Reagent (Sigma, Castle Hill, NSW, Australia) following the manufacturers protocol. All procedures were carried out using RNase-free reagents and consumables. Two micrograms of RNA were reverse transcribed into cDNA using Moloney-murine leukemia virus reverse transcriptase and random primers (Promega, Annandale, NSW, Australia) in a 20 μl reaction mixture. cDNA was then used as a template in quantitative PCR, using a Mastercycler ep realplex S (Eppendorf) and the fluorescent dye SYBR Green (Bio-Rad), following the manufacturer’s protocol. Briefly, each reaction (20 μl) contained 1x RealMasterMix, 1x SYBR green, 5 pmoles of primers and 1 μl of template. Amplification was carried out with 40 cycles of 94°C for 15 s and 60°C for 1 min. Gene expression was normalized to the housekeeper genes β-actin, GAPDH and cyclophilin A. All primers used are listed in Table 2. A no-template control was included for each PCR amplification assay. The level of expression for each gene was calculated using the comparative threshold cycle (Ct) value method using the formula 2^-ΔΔCt^ (where ΔΔCt = ΔCt sample – ΔCt reference).

**Table 2:** Primer sequences.

Gene	Sequence (5′-3′)	Product size (bp)
ABCA1	F: AACTCTACATCTCCCTTCCCG	123
	R: CTCCTGTCGCATGTCACTCC	
ABCA2	F: AGTGCTCAGCCTTCGTACAG	188
	R: AGGCGCGTACAGGATTTTGG	
ABCA3	F: ACGGTCCTGGAACTCTTCCT	187
	R: TGTGAGAAGGGATGTAGGCAA	
ABCA4	F: CGCTCAGGCAGAACCATCAT	102
	R: TGAGCAGTAGAGCCTTCCCT	
ABCA5	F: AGCCAAACAGCACATGTGGCGA	101
	R: AGACAGCCTCTGCCTCCTCCA	
ABCA6	F: GCTTCATTTCTCCCCACTTGTAT	102
	R: GCTGAATCTTGGAGACCCATCA	
ABCA7	F: CTAGCCGATGCCCGCACTGT	170
	R: GACGTCAGCAGCTCCGCGA	
ABCA8	F: ACCTGGGACGGGTAGATACAT	130
	R: CCCAAGACCTCTTTACCTGCC	
ABCA9	F: TAGCCCCTTTGCCTTCACTG	195
	R: TGTCCATATTCAGCGGGCAA	
ABCA10	F: GTTAAGGCGTGAAAGGAGAGC	168
	R: GGCGTCTTCGGGATTTGTTC	
ABCA13	F: GCAGAGGTTCTTGGGGGAAT	115
	R: CACTTCCAGTTCTTGGCCCT	
β-ACTIN	F: GAATTCTGGCCACGGCTGCTTCCAGCT	163
	R: AAGCTTTTTCGTGGATGCCACAGGACT	
CYCLOPHILIN A	F: AGGGTTCCTGCTTTCACAGA	211
	R: GTCTTGGCAGTGCAGATGAA	
GAPDH	F: AATGAAGGGGTCATTGATGG	108
	R: AAGGTGAAGGTCGGAGTCAA	
GFAP	F: TCCTGGAACAGCAAAACAAG	224
	R: CAGCCTCAGGTTGGTTTCAT	
MAP2	F: TTGGTGCCGAGTGAGAAGA	99
	R: GTCTGGCAGTGGTTGGTTAA	
TARDBP	F: AGCCAAGATGAGCCTTTGAGAA	94
	R: ACTGAGAGAAGAACTCCCGC	
TMEM119	F: CTGGCCTTTCTGCTGATGTTC	116
	R: TCACTCTGGTCCACGTACTTC	
TPPP	F: GAGCAGCGAGGAGGCCGTT	105
	R: GCCTCGACACTGTGGGCGAC	

Sequence of forward (F) and reverse (R) primers and product size is provided.

### Statistical analysis

All statistical analyses were performed using SPSS statistical software (IBM, Chicago, IL, United States). A multivariate general linear model analysis covarying for age, sex and PMI was used to determine differences in ABCA, TARDBP and neural subtype mRNA expression in FTLD-TDP compared to controls, with *post hoc* statistical significance set at *p* < 0.05. Partial correlations co-varying for age, sex and PMI were used to determine if alterations in ABCA expression were associated with alterations in neural subtype and/or TARDBP mRNA expression with statistical significance set at *p* < 0.05.

## Results

### Expression of ABCA transporters in FTLD-TDP brain

**Figure 1:**
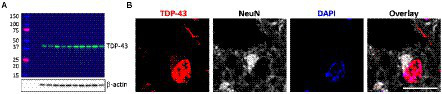
Verification of TDP-43 pathology in FTLD-TDP cases. **(A)** Western blotting of TDP-43 protein (43  kDa) in superior frontal cortex (SFC) in each FTLD-TDP (N = 10) cases; β-actin as a loading control; protein ladder (kDa). **(B)** Immunohistochemistry of neurons in FTLD-TDP SFC stained with TDP-43 (red), NeuN (grey), and DAPI (blue). Scale bar = 20 μm.

**Figure 2:**
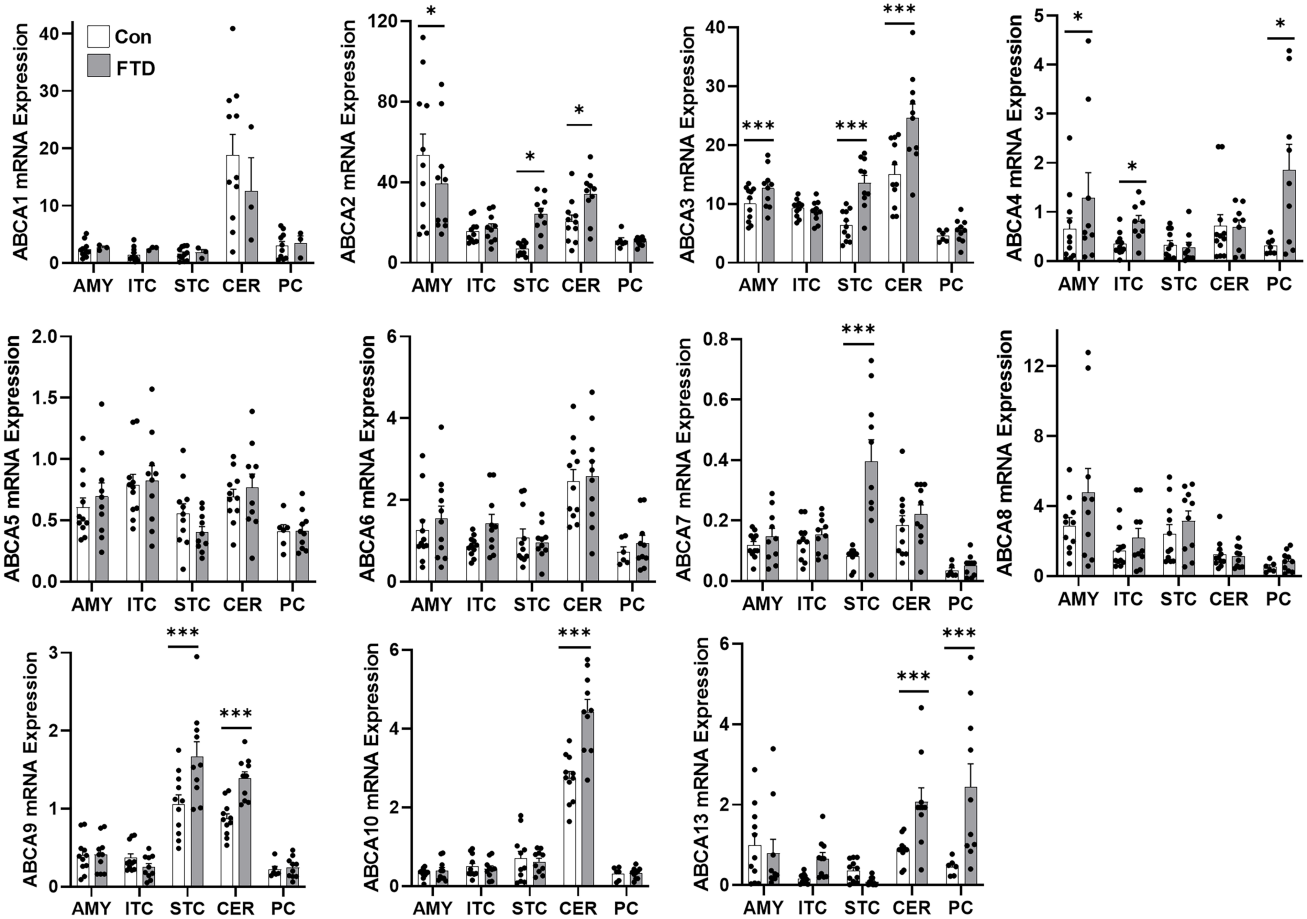
mRNA expression of ABCA transporters in FTLD-TDP and control brain. mRNA expression of ABCA transporters in amygdala (AMY), inferior temporal cortex (ITC), superior frontal cortex (SFC), cerebellum (CER) and parietal cortex (PC) in FTLD-TDP and control brain as measured by qPCR. Data represent mean and SE as error bars, *p < 0.05 and ***p < 0.001.

Firstly, TDP-43 pathology in FTLD-TDP cases was confirmed by western blotting (Figure 1A) and immunohistochemistry (Figure 1B). To determine if ABCA transporter expression was altered in FTLD-TDP, qPCR was performed to measure the overall relative expression of all ABCA genes in five brain regions, amygdala (affected early), inferior temporal cortex, superior frontal cortex, cerebellum and parietal cortex (affected late; Figure 2). ABCA2, ABCA3, ABCA4, ABCA7, ABCA9, ABCA10 and ABCA13 all demonstrated altered mRNA expression in specific regions of FTLD-TDP compared to controls. There were no significant changes to other transporters in any of the regions examined, while ABCA12 expression was undetectable in all five regions. ABCA2 was decreased 0.2-fold in the amygdala and increased in the superior frontal cortex (4-fold) and cerebellum (1.8-fold). ABCA3 was elevated in the amygdala (1.2-fold), superior frontal cortex (2.1-fold) and the cerebellum (1.6-fold). ABCA4 was elevated in the amygdala (2.1-fold), the inferior temporal cortex (3.9-fold) and the parietal cortex (4.8-fold). ABCA7 was increased 4.9-fold in the superior frontal cortex. ABCA9 was lower in the inferior temporal cortex (0.4-fold) but 1.5-fold higher in both the superior frontal cortex and cerebellum. ABCA10 was increased 1.6-fold in the cerebellum only. ABCA13 was elevated in the inferior temporal cortex (7-fold), cerebellum (2.6-fold) and the parietal cortex (4.5-fold).

### Expression of neural markers and TARDBP in FTLD-TDP brain

**Figure 3:**
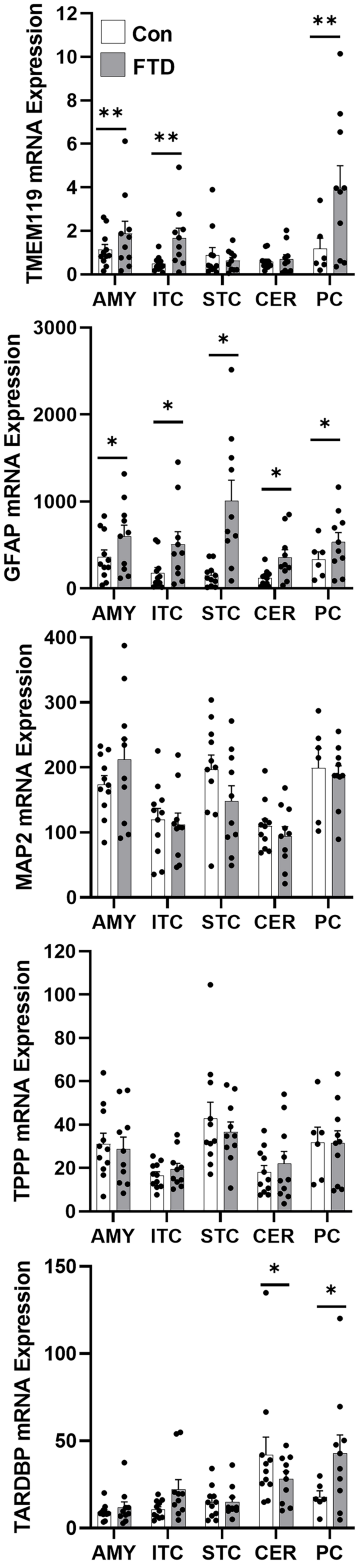
mRNA expression of TARDBP and neural markers in FTLD-TDP and control brain. mRNA expression of TARDBP and neural markers, TMEM119 (microglia), GFAP (astrocytes), MAP2 (neurons) and TPPP (oligodendrocytes) in amygdala (AMY), inferior temporal cortex (ITC), superior frontal cortex (SFC), cerebellum (CER) and parietal cortex (PC) in FTLD-TDP and control brain as measured by qPCR. Data represent mean and SE as error bars, *p < 0.05 and **p < 0.01.

To quantify mRNA expression of neural subtype markers, qPCR was performed for markers for neurons (MAP2), microglia (TMEM119), oligodendrocytes (TPPP) and astrocytes (GFAP) in all five brain regions (Figure 3). Additionally, TARDBP was also measured to evaluate TDP-43 expression in these brain regions. TMEM119, GFAP and TARDBP showed significant region-specific altered expression in FTLD-TDP compared to controls (Figure 3). TMEM119 was increased in the amygdala (1.9-fold), the inferior temporal cortex (4.4-fold) and the parietal cortex (3.7-fold). GFAP was elevated in all brain regions examined: 1.9-fold in the amygdala, 3.6-fold in the inferior temporal cortex, 8.8-fold in the superior frontal cortex, 4.6-fold in the cerebellum and 1.9-fold in the parietal cortex. TARDBP was significantly increased in the inferior temporal cortex (2.2-fold) and the parietal cortex (2.5-fold) and decreased in the cerebellum (0.3-fold).

### Correlations between ABCA transporters and neural markers

**Figure 4:**
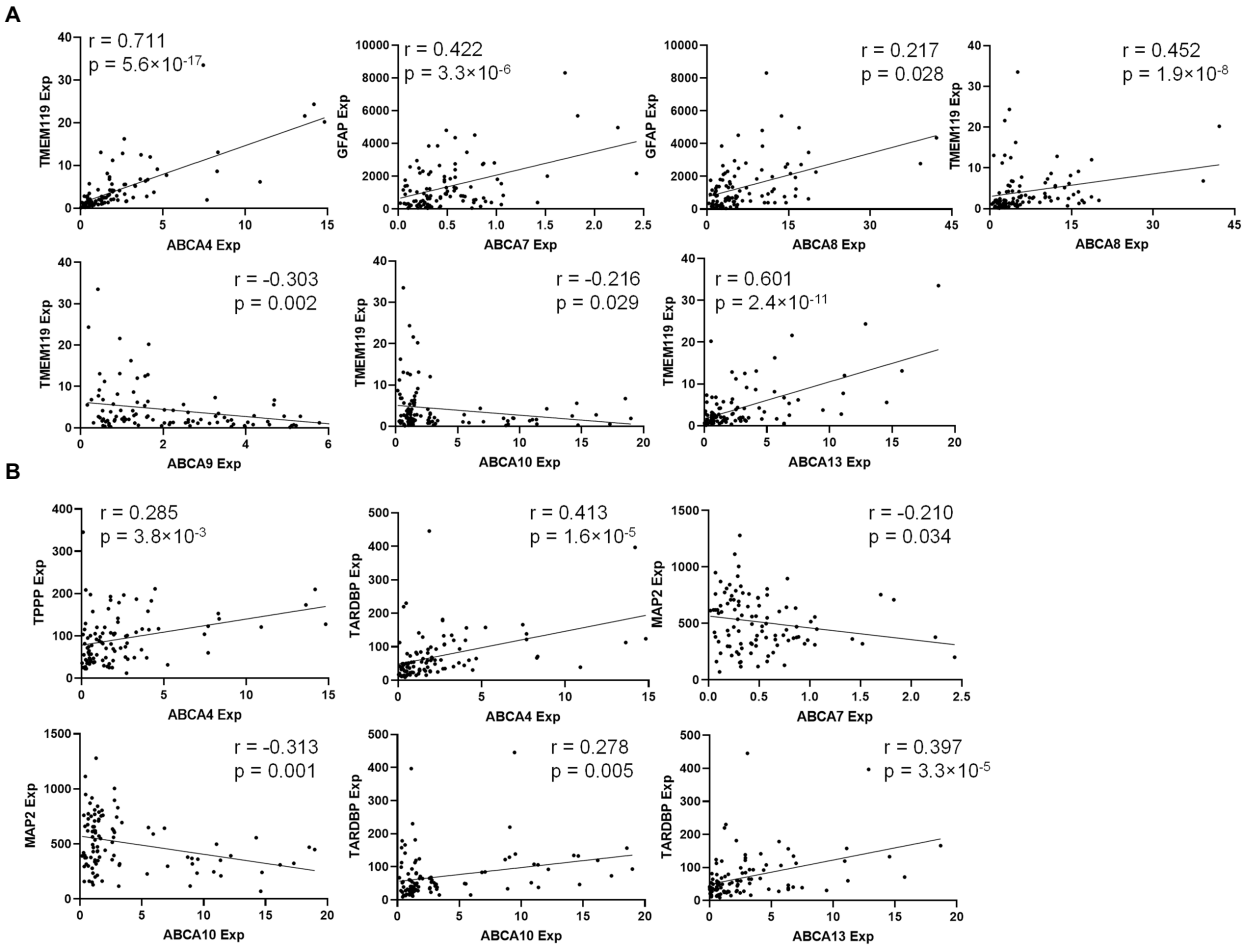
Correlation analysis of ABCA transporters with immune related neural markers and TARDBP. **(A)** A significant correlation between ABCA transporter expression and immune neural markers. **(B)** A significant correlation between ABCA transporter expression and non-immune neural markers.

A partial correlation analysis covarying for age, sex and PMI was performed to determine if there was a relationship between ABCA transporters, TARDBP and neural subtype markers: immune related neural markers (TMEM119 and GFAP; Figure 4A) and non-immune related neural markers (MAP2 and TPPP; Figure 4B). While correlation analysis was performed for all ABCA transporters, only ABCA transporters that showed significant expression changes in FTLD-TDP in Figure 2 are discussed here. ABCA4 correlated positively with TMEM119 (*r* = 0.711, *p* < 0.0001; Figure 4A), TPPP (*r* = 0.285, *p* < 0.01) and TARDBP (*r* = 0.413, *p* < 0.001; Figure 4B). ABCA7 levels correlated positively with GFAP (*r* = 0.442, *p* < 0.0001; Figure 4A) and negatively with MAP2 (*r* = −0.210, *p* < 0.05; Figure 4B). ABCA8 correlated positively to both GFAP (*r* = 0.442, *p* < 0.0001) and TMEM119 (*r* = 0.452, *p* < 0.0001; Figure 4A). ABCA9 correlated negatively with TMEM119 (*r* = −0.303, *p* < 0.002; Figure 4A). There is a weak negative correlation between ABCA10 and TMEM119 (*r* = −0.216, *p* < 0.05; Figure 4A) and MAP2 (*r* = −0.313, *p* = 0.001; Figure 4B) and a weak positive correlation with TARDBP (*r* = 0.278, *p* = 0.005; Figure 4B). ABCA13 correlated strongly with TMEM119 (*r* = 0.601, *p* < 0.001; Figure 4A) and TARDBP (*r* = 0.397, *p* < 0.001; Figure 4B).

## Discussion

ABCA transporters have been associated with numerous pathologies and are increasingly studied in relation to AD but there is limited knowledge on other neurodegenerative diseases, particularly those with TDP-43 pathology, such as FTLD-TDP. In this study, we provide a comprehensive analysis of ABCA transporter expression in the human brain across multiple brain regions. We report changes in ABCA transporter expression in FTLD-TDP compared to controls, with these changes correlating with expression of neural subtype markers, particularly those relating to inflammation (GFAP and TMEM119). This suggests that ABCA transporters may play a role in neuroinflammation in FTLD-TDP. Considering neuroinflammation is increasingly recognized as a hallmark of FTLD-TDP, further understanding the involvement of ABCA transporter dysregulation in neuroinflammation could provide insights into the aetiology of FTLD-TDP.

The expression of ABCA transporters in the FTLD-TDP was vastly different to that of normal brains, with abundant lipid dysregulation indicated by the upregulation of many of these ABCA lipid transporters. Currently, lipid dysregulation has not been well investigated in FTLD-TDP with only some recent studies examining lipid changes in blood of patients with frontotemporal dementia (FTD; Kim et al., 2018; Murley et al., 2020; Phan et al., 2020). In a neuronal model of FTD tauopathy, dysregulation of the lipid phosphatidylserine was shown to trigger microglial phagocytosis of affected neurons (Brelstaff et al., 2018).

The role of ABCA transporters in lipid dysregulation and neurodegeneration has been studied in greater detail in AD, where ABCA7 and ABCA2 have been implicated in the aetiology of AD. ABCA2 overexpression increases expression of genes associated with neurodegeneration and ABCA2 knockdown reduces amyloid-β production (Davis and Tew, 2018). Loss-of-function mutations in ABCA7 lead to impaired amyloid-β clearance and dysregulated macrophage phagocytosis (Aikawa et al., 2018). The maintenance of ABCA2 and ABCA7 protein levels is associated with cell protection by removal of toxic lipids (Chen et al., 2004; Lyssenko and Pratico, 2021), particularly neuronal ROS peroxidated lipid which is sequestrated into glial lipid droplets, delaying neurotoxicity (Moulton et al., 2021). Our results show ABCA2 and ABCA7 upregulation in FTLD-TDP in a region-specific manner, with significant increases in ABCA7 expression in the superior frontal cortex (Figure 2), a region which usually sustains substantial tissue loss. We also show a strong positive association between ABCA7 and GFAP levels, an astrocyte marker, consistent with the concept that the increase in ABCA7 is largely in glia that could assist with peroxidated lipid sequestration in astrocytes (Moulton et al., 2021). The elevated levels of ABCA2 and ABCA7 in FTLD-TDP reported here could be critical to compensatory mechanisms. Interestingly, rare variants of the *ABCA7* gene have been identified in two sporadic cases of FTD (Ciani et al., 2019) and a homozygous loss-of-function *ABCA7* variant was also identified, suggesting *ABCA7* as a candidate gene for monogenic FTD (Wagner et al., 2021). A partial deletion in the *ABCA7* gene and a variant in the *GRN* gene has been found in a patient with semantic variant of primary progressive aphasia (Antonell et al., 2020), one of the clinical phenotypes associated with FTLD-TDP. Loss-of-function mutations in *ABCA7* are also prevalent in AD (Hollingworth et al., 2011; Steinberg et al., 2015) with evidence of TDP-43 positive neurofibrillary tangles and neuronal cytoplasmic inclusions in these cases (Van den Bossche et al., 2016) despite lower protein levels. This would be consistent with ABCA7 delaying neurotoxicity through glial mechanisms (Moulton et al., 2021), and that dysregulation of that mechanism and increases in ABCA2 induce neuronal TDP-43 deposition potentially through protein condensation phenomena (Vendruscolo, 2022). The regional selectivity in FTLD-TDP warrants further research in concert with the regional involvement of ABCA transporters in glia and neurons in FTLD-TDP.

In contrast, ABCA1, ABCA5 and ABCA8 levels were not significantly altered in FTLD-TDP. Physiologically, ABCA1 plays a major role in cholesterol efflux and regulates intracellular cholesterol levels. ABCA1 is critical in physiological brain function where it loads newly synthesised cholesterol from astrocytes onto apoE/apoA-I to transport cholesterol to neurons and myelin (Zhao et al., 2017), a mechanism which does not appear to be disrupted in FTLD-TDP. ABCA5 has also been implicated as a cholesterol efflux transporter (Fu et al., 2015) while ABCA8 may impact oligodendroglial functions (Bleasel et al., 2013). Our results show that the levels of ABCA8 positively correlated with increased astrocytic and microglial markers in FTLD-TDP (Figure 4A). As loss of ABCA8 reduces oligodendroglial precursors and mature oligodendrocytes (Liu et al., 2022) and its expression in human prefrontal cortex associates with myelination (Kim et al., 2013), its positive association to increased astrocytic GFAP (most dominant in superior frontal cortex) may suggest additional compensation of cortical oligodendrocytes to FTLD-TDP.

Our results also identify dysfunction of numerous ABCA transporters in FTLD-TDP that have not previously been implicated in neurodegeneration. Very little is known about ABCA3, ABCA9 and ABCA10. ABCA3 has been pathologically linked to surfactant deficiency, a lung disorder relating to aberrant lipid transport that is fatal in newborns (Shulenin et al., 2004) while there is very little knowledge on the physiological and pathological functions of ABCA9 and ABCA10. ABCA4 is associated with autosomal recessive Stargardt macular dystrophy (Sun et al., 2000). It is thought to be primarily associated with the retina with a murine model of Stargardt macular dystrophy that contained double knockout Abca4^−/−^ and Rdh8^−/−^ exhibiting reduced inflammation (Kohno et al., 2013). Some evidence suggest that structural changes to the brain are associated with Stargardt’s disease. In one study, imaging analyses showed a significant grey matter loss bilaterally in the occipital cortices and in the fronto-orbital cortices, and reductions in fractional anisotropy in the supratentorial white matter regions (Olivo et al., 2015). ABCA13 is expressed on the photoreceptor outer segments and functions in the visual cycle. It facilitates the transport of retinylidene-phosphatidylethanolamine or phosphatidylethanolamine from the lumen to the cytoplasm of the disc membrane by acting as a flippase. In Stargardt macular dystrophy, the loss of this function led to disruption in the transport of these two molecules (Sun et al., 2000; Tsybovsky et al., 2010; Quazi et al., 2012).

Our results show that ABCA13 was the most dysregulated ABCA transporter globally in FTLD-TDP. ABCA13 has been studied mainly in terms of mental conditions, such as schizophrenia, bipolar disorder or depression (Knight et al., 2009). It is downregulated in schizophrenia, bipolar disorder, major depression (Knight et al., 2009; Dwyer et al., 2011; Chen et al., 2017) and dementia with Lewy bodies (Rajkumar et al., 2019), and upregulated in glioblastoma (Drean et al., 2018) and adenocarcinoma (Araujo et al., 2016). In a recent study, ABCA13 was shown to be associated with synaptic function in autism spectrum disorder (Kimura et al., 2022). Its downregulation results in impaired protein subcellular localisation and cholesterol trafficking (Nakato et al., 2021). The downregulation of ABCA13 in these psychiatric conditions is in contrast to the upregulation of ABCA13 (and ABCA4) in FTLD-TDP, implying different roles for these ABCA transporters in FTLD-TDP. The expression level of both ABCA4 and ABCA13 strongly correlated with the microglial cell markers TMEM119 and to TARDBP expression, suggesting a potential role for these ABCA transporters in neuroinflammation in FTLD-TDP. Since neuroinflammation is widely considered as a hallmark of neurodegenerative diseases and its role in the aetiology of FTLD is of increasing interest (Bright et al., 2019), the relationship between neuroinflammation, TARDBP and ABCA4 and ABCA13 should be further examined to determine if these ABCA transporters exhibit neuroprotective or more neurotoxic roles. Interestingly, there have been recent reports of TDP-43 inhibition of cholesterol biosynthesis through the master regulator of cholesterol homeostasis, sterol regulatory element-binding protein 2 (Egawa et al., 2022), particularly in the ubiquitous oligodendrocytes harbouring TDP-3 inclusion pathology in FTLD-TDP (Ho et al., 2021).

The strength of this current study is the comprehensive coverage of the expression of all members of the ABCA transporter family. This is important as most of the ABCA transporters have not been investigated in the context of FTLD-TDP, let alone FTLD-TDP brain, despite the fact that lipid dysregulation is intrinsically linked to neurodegeneration. Another strength of this study is the analysis of multiple regions of the brain that encompasses different stages of FTLD-TDP. This is important as lipid dysregulation changes with disease progression that would impact the response of the ABCA transporters. This study could have been further strengthened with the addition of protein expression data, although reliable antibodies against most of the ABCA transporters are not available for human protein lysates. Also, further research is required to determine regional and cell-types specific changes in ABCA transporters and their effect on FTLD-TDP tissue changes.

We provide new data characterising the expression of ABCA transporters in five brain regions affected at different stages of FTLD-TDP, correlating changes in ABCA transporters with expression of TARDBP and cell subtype markers. Data suggestive of cell type specific increases in ABCA transporters in FTLD-TDP include ABCA7 in astrocytes and ABCA4 and ABCA13 in microglia, with these microglia increases associated with increased TARDBP expression. Regulation of these transporters in the cell types identified has not been previously determined, requiring further research. However, the identification of widespread dysregulation of particular ABCA transporters, some not previously linked to neurodegenerative diseases, implies new and important physiological roles of these transporters in association with FTLD-TDP.

## Data availability statement

The original contributions presented in the study are included in the article/supplementary material, further inquiries can be directed to the corresponding author.

## Author contributions

JK carried out RNA extraction and qPCR, analyzed the data, and wrote the manuscript. HL provided expert advice and revised the manuscript. SB prepared tissue lysates and carried out western blotting. YF carried out immunohistochemical analysis. GH provided expert advice and revised the manuscript. WK conceived, developed, and supervised the project, analyzed the data, and wrote the manuscript. All authors contributed to the article and approved the submitted version.

## Funding

This work was supported by funding to ForeFront, a collaborative research group dedicated to the study of frontotemporal dementia and motor neuron disease, from the National Health and Medical Research Council of Australia (NHMRC) grants (#1132524 and #1095127). G.M.H. is a NHMRC Senior Leadership Fellow (#1176607). Tissues were received from the New South Wales Brain Tissue Resource Centre at the University of Sydney and the Sydney Brain Bank at Neuroscience Research Australia, which are supported by The University of New South Wales, Neuroscience Research Australia and Schizophrenia Research Institute. Research reported in this publication was supported by the National Institute on Alcohol Abuse and Alcoholism of the National Institutes of Health under Award Number R28AA012725. The content is solely the responsibility of the authors and does not necessarily represent the official views of the National Institutes of Health.

## Conflict of interest

The authors declare that the research was conducted in the absence of any commercial or financial relationships that could be construed as a potential conflict of interest.

## Publisher’s note

All claims expressed in this article are solely those of the authors and do not necessarily represent those of their affiliated organizations, or those of the publisher, the editors and the reviewers. Any product that may be evaluated in this article, or claim that may be made by its manufacturer, is not guaranteed or endorsed by the publisher.

## References

[ref1] Aikawa T. Holm M. L. Kanekiyo T. (2018). ABCA7 and pathogenic pathways of Alzheimer's disease. Brain Sci. 8:0027. doi: 10.3390/brainsci8020027, PMID: 29401741 PMC5836046

[ref2] Allikmets R. Shroyer N. F. Singh N. Seddon J. M. Lewis R. A. Bernstein P. S.. (1997). Mutation of the Stargardt disease gene (ABCR) in age-related macular degeneration. Science 277, 1805–1807. doi: 10.1126/science.277.5333.1805 9295268

[ref3] Amador-Ortiz C. Lin W. L. Ahmed Z. Personett D. Davies P. Duara R.. (2007). TDP-43 immunoreactivity in hippocampal sclerosis and Alzheimer's disease. Ann. Neurol. 61, 435–445. doi: 10.1002/ana.21154, PMID: 17469117 PMC2677204

[ref4] Antonell A. Ramos-Campoy O. Balasa M. Borrego-Ecija S. Montagut N. Falgas N.. (2020). An ABCA7 partial deletion and a GRN variant in a semantic variant of primary progressive aphasia patient. Alzheimers Dement. 16:e042483. doi: 10.1002/alz.042483

[ref5] Araujo T. M. Seabra A. D. Lima E. M. Assumpcao P. P. Montenegro R. C. Demachki S.. (2016). Recurrent amplification of RTEL1 and ABCA13 and its synergistic effect associated with clinicopathological data of gastric adenocarcinoma. Mol. Cytogenet. 9:52. doi: 10.1186/s13039-016-0260-x, PMID: 27366209 PMC4928298

[ref6] Bevan-Jones W. R. Cope T. E. Jones P. S. Kaalund S. S. Passamonti L. Allinson K.. (2020). Neuroinflammation and protein aggregation co-localize across the frontotemporal dementia spectrum. Brain 143, 1010–1026. doi: 10.1093/brain/awaa033, PMID: 32179883 PMC7089669

[ref7] Bleasel J. M. Hsiao J. H. Halliday G. M. Kim W. S. (2013). Increased expression of ABCA8 in multiple system atrophy brain is associated with changes in pathogenic proteins. J. Parkinsons Dis. 3, 331–339. doi: 10.3233/JPD-130203, PMID: 23948991

[ref8] Brelstaff J. Tolkovsky A. M. Ghetti B. Goedert M. Spillantini M. G. (2018). Living neurons with tau filaments aberrantly expose Phosphatidylserine and are Phagocytosed by microglia. Cell Rep. 24:e1934, 1939–1948. doi: 10.1016/j.celrep.2018.07.072 PMC6161320 30134156

[ref9] Bright F. Werry E. L. Dobson-Stone C. Piguet O. Ittner L. M. Halliday G. M.. (2019). Neuroinflammation in frontotemporal dementia. Nat. Rev. Neurol. 15, 540–555. doi: 10.1038/s41582-019-0231-z 31324897

[ref10] Brooks-Wilson A. Marcil M. Clee S. M. Zhang L. H. Roomp K. Van Dam M.. (1999). Mutations in ABC1 in Tangier disease and familial high-density lipoprotein deficiency. Nat. Genet. 22, 336–345. doi: 10.1038/11905, PMID: 10431236

[ref11] Cairns N. J. Bigio E. H. Mackenzie I. R. Neumann M. Lee V. M. Hatanpaa K. J.. (2007). Neuropathologic diagnostic and nosologic criteria for frontotemporal lobar degeneration: consensus of the consortium for Frontotemporal lobar degeneration. Acta Neuropathol. 114, 5–22. doi: 10.1007/s00401-007-0237-2 17579875 PMC2827877

[ref12] Chen J. Khan R. A. W. Wang M. He K. Wang Q. Li Z.. (2017). Association between the variability of the ABCA13 gene and the risk of major depressive disorder and schizophrenia in the Han Chinese population. World J. Biol. Psychiatry 18, 550–556. doi: 10.1080/15622975.2016.1245442, PMID: 27712136

[ref13] Chen Z. J. Vulevic B. Ile K. E. Soulika A. Davis W. Jr. Reiner P. B.. (2004). Association of ABCA2 expression with determinants of Alzheimer's disease. FASEB J. 18, 1129–1131. doi: 10.1096/fj.03-1490fje, PMID: 15155565

[ref14] Ciani M. Bonvicini C. Scassellati C. Carrara M. Maj C. Fostinelli S.. (2019). The missing heritability of sporadic Frontotemporal dementia: new insights from rare variants in neurodegenerative candidate genes. Int. J. Mol. Sci. 20:3903. doi: 10.3390/ijms20163903, PMID: 31405128 PMC6721049

[ref15] Davis W. Tew K. D. (2018). ATP-binding cassette transporter-2 (ABCA2) as a therapeutic target. Biochem. Pharmacol. 151, 188–200. doi: 10.1016/j.bcp.2017.11.018, PMID: 29223352 PMC5899931

[ref16] Drean A. Rosenberg S. Lejeune F. X. Goli L. Nadaradjane A. A. Guehennec J.. (2018). ATP binding cassette (ABC) transporters: expression and clinical value in glioblastoma. J. Neuro-Oncol. 138, 479–486. doi: 10.1007/s11060-018-2819-3, PMID: 29520610

[ref17] Dwyer S. Williams H. Jones I. Jones L. Walters J. Craddock N.. (2011). Investigation of rare non-synonymous variants at ABCA13 in schizophrenia and bipolar disorder. Mol. Psychiatry 16, 790–791. doi: 10.1038/mp.2011.2, PMID: 21283083

[ref18] Egawa N. Izumi Y. Suzuki H. Tsuge I. Fujita K. Shimano H.. (2022). TDP-43 regulates cholesterol biosynthesis by inhibiting sterol regulatory element-binding protein 2. Sci. Rep. 12:7988. doi: 10.1038/s41598-022-12133-4, PMID: 35568729 PMC9107471

[ref19] Fu Y. Hsiao J. H. Paxinos G. Halliday G. M. Kim W. S. (2015). ABCA5 regulates amyloid-beta peptide production and is associated with Alzheimer's disease neuropathology. J. Alzheimers Dis. 43, 857–869. doi: 10.3233/JAD-141320, PMID: 25125465

[ref20] Fu Y. Hsiao J. H. Paxinos G. Halliday G. M. Kim W. S. (2016). ABCA7 mediates phagocytic clearance of amyloid-beta in the brain. J. Alzheimers Dis. 54, 569–584. doi: 10.3233/JAD-160456, PMID: 27472885

[ref21] Ho W. Y. Chang J. C. Lim K. Cazenave-Gassiot A. Nguyen A. T. Foo J. C.. (2021). TDP-43 mediates SREBF2-regulated gene expression required for oligodendrocyte myelination. J. Cell Biol. 220:e201910213. doi: 10.1083/jcb.201910213 34347016 PMC8348376

[ref22] Hollingworth P. Harold D. Sims R. Gerrish A. Lambert J. C. Carrasquillo M. M.. (2011). Common variants at ABCA7, MS4A6A/MS4A4E, EPHA1, CD33 and CD2AP are associated with Alzheimer's disease. Nat. Genet. 43, 429–435. doi: 10.1038/ng.803, PMID: 21460840 PMC3084173

[ref23] Hyman B. T. Phelps C. H. Beach T. G. Bigio E. H. Cairns N. J. Carrillo M. C.. (2012). National Institute on Aging-Alzheimer's Association guidelines for the neuropathologic assessment of Alzheimer's disease. Alzheimers Dement. 8, 1–13. doi: 10.1016/j.jalz.2011.10.007, PMID: 22265587 PMC3266529

[ref24] Josephs K. A. Murray M. E. Whitwell J. L. Parisi J. E. Petrucelli L. Jack C. R.. (2014). Staging TDP-43 pathology in Alzheimer's disease. Acta Neuropathol. 127, 441–450. doi: 10.1007/s00401-013-1211-9, PMID: 24240737 PMC3944799

[ref25] Karasinska J. M. De Haan W. Franciosi S. Ruddle P. Fan J. Kruit J. K.. (2013). ABCA1 influences neuroinflammation and neuronal death. Neurobiol. Dis. 54, 445–455. doi: 10.1016/j.nbd.2013.01.018, PMID: 23376685

[ref26] Katzeff J. S. Kim W. S. (2021). ATP-binding cassette transporters and neurodegenerative diseases. Essays Biochem. 65, 1013–1024. doi: 10.1042/EBC20210012 34415015

[ref27] Kelsell D. P. Norgett E. E. Unsworth H. Teh M. T. Cullup T. Mein C. A.. (2005). Mutations in ABCA12 underlie the severe congenital skin disease harlequin ichthyosis. Am. J. Hum. Genet. 76, 794–803. doi: 10.1086/429844, PMID: 15756637 PMC1199369

[ref28] Kim W. S. Hsiao J. H. Bhatia S. Glaros E. N. Don A. S. Tsuruoka S.. (2013). ABCA8 stimulates sphingomyelin production in oligodendrocytes. Biochem. J. 452, 401–410. doi: 10.1042/BJ20121764, PMID: 23560799

[ref29] Kim W. S. Jary E. Pickford R. He Y. Ahmed R. M. Piguet O.. (2018). Lipidomics analysis of behavioral variant Frontotemporal dementia: A scope for biomarker development. Front. Neurol. 9:104. doi: 10.3389/fneur.2018.00104, PMID: 29541056 PMC5835505

[ref30] Kimura H. Nakatochi M. Aleksic B. Guevara J. Toyama M. Hayashi Y.. (2022). Exome sequencing analysis of Japanese autism spectrum disorder case-control sample supports an increased burden of synaptic function-related genes. Transl. Psychiatry 12:265. doi: 10.1038/s41398-022-02033-6, PMID: 35811316 PMC9271461

[ref31] Knight H. M. Pickard B. S. Maclean A. Malloy M. P. Soares D. C. Mcrae A. F.. (2009). A cytogenetic abnormality and rare coding variants identify ABCA13 as a candidate gene in schizophrenia, bipolar disorder, and depression. Am. J. Hum. Genet. 85, 833–846. doi: 10.1016/j.ajhg.2009.11.003, PMID: 19944402 PMC2790560

[ref32] Kohno H. Chen Y. Kevany B. M. Pearlman E. Miyagi M. Maeda T.. (2013). Photoreceptor proteins initiate microglial activation via toll-like receptor 4 in retinal degeneration mediated by all-trans-retinal. J. Biol. Chem. 288, 15326–15341. doi: 10.1074/jbc.M112.448712, PMID: 23572532 PMC3663552

[ref33] Liu Y. Castano D. Girolamo F. Trigueros-Motos L. Bae H. G. Neo S. P.. (2022). Loss of ABCA8B decreases myelination by reducing oligodendrocyte precursor cells in mice. J. Lipid Res. 63:100147. doi: 10.1016/j.jlr.2021.100147, PMID: 34752805 PMC8953628

[ref34] Lupton M. K. Proitsi P. Lin K. Hamilton G. Daniilidou M. Tsolaki M.. (2014). The role of ABCA1 gene sequence variants on risk of Alzheimer's disease. J. Alzheimers Dis. 38, 897–906. doi: 10.3233/JAD-131121, PMID: 24081377

[ref35] Lyssenko N. N. Pratico D. (2021). ABCA7 and the altered lipidostasis hypothesis of Alzheimer's disease. Alzheimers Dement. 17, 164–174. doi: 10.1002/alz.12220, PMID: 33336544 PMC7986801

[ref36] Mace S. Cousin E. Ricard S. Genin E. Spanakis E. Lafargue-Soubigou C.. (2005). ABCA2 is a strong genetic risk factor for early-onset Alzheimer's disease. Neurobiol. Dis. 18, 119–125. doi: 10.1016/j.nbd.2004.09.011, PMID: 15649702

[ref37] Mackenzie I. R. Neumann M. Bigio E. H. Cairns N. J. Alafuzoff I. Kril J.. (2010). Nomenclature and nosology for neuropathologic subtypes of frontotemporal lobar degeneration: an update. Acta Neuropathol. 119, 1–4. doi: 10.1007/s00401-009-0612-2, PMID: 19924424 PMC2799633

[ref38] Montine T. J. Phelps C. H. Beach T. G. Bigio E. H. Cairns N. J. Dickson D. W.. (2012). National Institute on Aging-Alzheimer’s Association guidelines for the neuropathologic assessment of Alzheimer's disease: a practical approach. Acta Neuropathol. 123, 1–11. doi: 10.1007/s00401-011-0910-3, PMID: 22101365 PMC3268003

[ref39] Moulton M. J. Barish S. Ralhan I. Chang J. Goodman L. D. Harland J. G.. (2021). Neuronal ROS-induced glial lipid droplet formation is altered by loss of Alzheimer's disease-associated genes. Proc. Natl. Acad. Sci. U. S. A. 118:118. doi: 10.1073/pnas.2112095118 PMC8719885 34949639

[ref40] Murley A. G. Jones P. S. Coyle Gilchrist I. Bowns L. Wiggins J. Tsvetanov K. A.. (2020). Metabolomic changes associated with frontotemporal lobar degeneration syndromes. J. Neurol. 267, 2228–2238. doi: 10.1007/s00415-020-09824-1, PMID: 32277260 PMC7359154

[ref41] Murphy K. E. Cottle L. Gysbers A. M. Cooper A. A. Halliday G. M. (2013). ATP13A2 (PARK9) protein levels are reduced in brain tissue of cases with Lewy bodies. Acta Neuropathol. Commun. 1:11. doi: 10.1186/2051-5960-1-11, PMID: 24252509 PMC4046687

[ref42] Nakato M. Shiranaga N. Tomioka M. Watanabe H. Kurisu J. Kengaku M.. (2021). ABCA13 dysfunction associated with psychiatric disorders causes impaired cholesterol trafficking. J. Biol. Chem. 296:100166. doi: 10.1074/jbc.RA120.015997, PMID: 33478937 PMC7948424

[ref43] Obrien J. S. Sampson E. L. (1965). Lipid composition of the normal human brain: gray matter, white matter, and myelin. J. Lipid Res. 6, 537–544. doi: 10.1016/S0022-2275(20)39619-X, PMID: 5865382

[ref44] Olivo G. Melillo P. Cocozza S. D'alterio F. M. Prinster A. Testa F.. (2015). Cerebral involvement in Stargardt's disease: A VBM and TBSS study. Invest. Ophthalmol. Vis. Sci. 56, 7388–7397. doi: 10.1167/iovs.15-16899, PMID: 26574798

[ref45] Phan K. He Y. Fu Y. Dzamko N. Bhatia S. Gold J.. (2021). Pathological manifestation of human endogenous retrovirus K in frontotemporal dementia. Commun Med (Lond) 1:60. doi: 10.1038/s43856-021-00060-w 35083468 PMC8788987

[ref46] Phan K. He Y. Pickford R. Bhatia S. Katzeff J. S. Hodges J. R.. (2020). Uncovering pathophysiological changes in frontotemporal dementia using serum lipids. Sci. Rep. 10:3640. doi: 10.1038/s41598-020-60457-w, PMID: 32107421 PMC7046653

[ref47] Piehler A. P. Ozcurumez M. Kaminski W. E. (2012). A-subclass ATP-binding cassette proteins in brain lipid homeostasis and Neurodegeneration. Front. Psych. 3:17. doi: 10.3389/fpsyt.2012.00017 PMC3293240 22403555

[ref48] Quazi F. Lenevich S. Molday R. S. (2012). ABCA4 is an N-retinylidene-phosphatidylethanolamine and phosphatidylethanolamine importer. Nat. Commun. 3:925. doi: 10.1038/ncomms1927, PMID: 22735453 PMC3871175

[ref49] Rabinovici G. D. Miller B. L. (2010). Frontotemporal lobar degeneration: epidemiology, pathophysiology, diagnosis and management. CNS Drugs 24, 375–398. doi: 10.2165/11533100-000000000-00000, PMID: 20369906 PMC2916644

[ref50] Rajkumar A. P. Bidkhori G. Shoaie S. Clarke E. Morrin H. Hye A.. (2019). Postmortem cortical Transcriptomics of Lewy body dementia reveal mitochondrial dysfunction and lack of Neuroinflammation. Am. J. Geriatr. Psychiatry 28, 75–86. doi: 10.1016/j.jagp.2019.06.007 31327631

[ref51] Sakae N. Liu C. C. Shinohara M. Frisch-Daiello J. Ma L. Yamazaki Y.. (2016). ABCA7 deficiency accelerates amyloid-beta generation and Alzheimer's neuronal pathology. J. Neurosci. 36, 3848–3859. doi: 10.1523/JNEUROSCI.3757-15.2016, PMID: 27030769 PMC4812140

[ref52] Shulenin S. Nogee L. M. Annilo T. Wert S. E. Whitsett J. A. Dean M. (2004). ABCA3 gene mutations in newborns with fatal surfactant deficiency. N. Engl. J. Med. 350, 1296–1303. doi: 10.1056/NEJMoa032178 15044640

[ref53] Steinberg S. Stefansson H. Jonsson T. Johannsdottir H. Ingason A. Helgason H.. (2015). Loss-of-function variants in ABCA7 confer risk of Alzheimer's disease. Nat. Genet. 47, 445–447. doi: 10.1038/ng.3246, PMID: 25807283

[ref54] Sun H. Smallwood P. M. Nathans J. (2000). Biochemical defects in ABCR protein variants associated with human retinopathies. Nat. Genet. 26, 242–246. doi: 10.1038/79994, PMID: 11017087

[ref55] Tan R. H. Shepherd C. E. Kril J. J. Mccann H. Mcgeachie A. Mcginley C.. (2013). Classification of FTLD-TDP cases into pathological subtypes using antibodies against phosphorylated and non-phosphorylated TDP43. Acta Neuropathol. Commun. 1:33. doi: 10.1186/2051-5960-1-33, PMID: 24252630 PMC4046675

[ref56] Trageser K. J. Smith C. Herman F. J. Ono K. Pasinetti G. M. (2019). Mechanisms of immune activation by c9orf72-expansions in amyotrophic lateral sclerosis and Frontotemporal dementia. Front. Neurosci. 13:1298. doi: 10.3389/fnins.2019.01298, PMID: 31920478 PMC6914852

[ref57] Tsybovsky Y. Molday R. S. Palczewski K. (2010). The ATP-binding cassette transporter ABCA4: structural and functional properties and role in retinal disease. Adv. Exp. Med. Biol. 703, 105–125. doi: 10.1007/978-1-4419-5635-4_8, PMID: 20711710 PMC2930353

[ref58] Van Den Bossche T. Sleegers K. Cuyvers E. Engelborghs S. Sieben A. De Roeck A.. (2016). Phenotypic characteristics of Alzheimer patients carrying an ABCA7 mutation. Neurology 86, 2126–2133. doi: 10.1212/WNL.0000000000002628, PMID: 27037232 PMC4917260

[ref59] Vendruscolo M. (2022). Lipid homeostasis and its links with protein Misfolding diseases. Front. Mol. Neurosci. 15:829291. doi: 10.3389/fnmol.2022.829291, PMID: 35401104 PMC8990168

[ref60] Wagner M. Lorenz G. Volk A. E. Brunet T. Edbauer D. Berutti R.. (2021). Clinico-genetic findings in 509 frontotemporal dementia patients. Mol. Psychiatry 26, 5824–5832. doi: 10.1038/s41380-021-01271-2, PMID: 34561610 PMC8758482

[ref61] Zhao Y. Hou D. Feng X. Lin F. Luo J. (2017). Role of ABC transporters in the pathology of Alzheimer's disease. Rev. Neurosci. 28, 155–159. doi: 10.1515/revneuro-2016-0060 27997355

